# Dynamic contrast-enhanced magnetic resonance imaging for head and neck cancers

**DOI:** 10.1038/sdata.2018.8

**Published:** 2018-02-13

**Authors:** Joint Head and Neck Radiotherapy-MRI Development Cooperative, Hesham Elhalawani, Rachel B. Ger, Abdallah S. R. Mohamed, Musaddiq J. Awan, Yao Ding, Kimberly Li, Xenia J. Fave, Andrew L. Beers, Brandon Driscoll, David A. Hormuth II, Petra J. van Houdt, Renjie He, Shouhao Zhou, Kelsey B. Mathieu, Heng Li, Catherine Coolens, Caroline Chung, James A. Bankson, Wei Huang, Jihong Wang, Vlad C. Sandulache, Stephen Y. Lai, Rebecca M. Howell, R Jason Stafford, Thomas E. Yankeelov, Uulke A. van der Heide, Steven J. Frank, Daniel P. Barboriak, John D. Hazle, Laurence E. Court, Jayashree Kalpathy-Cramer, Clifton D. Fuller

**Affiliations:** 1 Department of Radiation Oncology, The University of Texas MD Anderson Cancer Center, Houston, Texas 77030, USA; 2 Department of Radiation Physics, The University of Texas MD Anderson Cancer Center, Houston, Texas 77030, USA; 3 The University of Texas MD Anderson Cancer Center UTHealth Graduate School of Biomedical Sciences, Houston, Texas 77030, USA; 4 Department of Clinical Oncology and Nuclear Medicine, Faculty of Medicine, University of Alexandria, Alexandria, Egypt 21526; 5 Case Western Reserve University, Cleveland, OH 44106, USA; 6 University Hospitals, Cleveland, OH 44106, USA; 7 Department of Imaging Physics, The University of Texas MD Anderson Cancer Center, Houston, Texas 77030, USA; 8 Advanced Imaging Research Center, Knight Cancer Institute, Oregon Health & Science University, Portland, Oregon 97007, USA; 9 The International School of Beaverton, Beaverton, Oregon 97007, USA; 10 Athinoula A. Martinos Center for Biomedical Imaging, Massachusetts General Hospital/Division of Health Sciences & Technology, Massachusetts Institute of Technology, Charlestown, Massachusetts 02129, USA; 11 Techna Institute, University Health Network, Toronto, Ontario, Canada M5G 1L5; 12 Departments of Biomedical Engineering and Internal Medicine, Institute for Computational Engineering and Sciences, Livestrong Cancer Institutes, The University of Texas, Austin, Texas 78712, USA; 13 Department of Radiation Oncology, The Netherlands Cancer Institute, Amsterdam, CX 1066 The Netherlands; 14 United Imaging Healthcare America, Houston, Texas 77054, USA; 15 Department of Biostatistics, The University of Texas MD Anderson Cancer Center, Houston, Texas 77030, USA; 16 Radiation Medicine Program, Princess Margaret Cancer Centre, University Health Network, Toronto, Ontario, Canada M5G 2M9; 17 Department of Radiation Oncology, University of Toronto, Toronto, Ontario, Canada M5S 3E2; 18 Department of Otolaryngology Head and Neck Surgery, Baylor College of Medicine, Houston, Texas 77030, USA; 19 Department of Head and Neck Surgery, The University of Texas MD Anderson Cancer Center, Houston, Texas 77030, USA; 20 Department of Molecular and Cellular Oncology, The University of Texas MD Anderson Cancer Center, Houston, Texas 77030, USA; 21 Department of Radiology, Duke University Medical Center, Durham, North Carolina, USA

**Keywords:** Oral cancer, Predictive markers, Cancer imaging

## Abstract

Dynamic myraidpro contrast-enhanced magnetic resonance imaging (DCE-MRI) has been correlated with prognosis in head and neck squamous cell carcinoma as well as with changes in normal tissues. These studies implement different software, either commercial or in-house, and different scan protocols. Thus, the generalizability of the results is not confirmed. To assist in the standardization of quantitative metrics to confirm the generalizability of these previous studies, this data descriptor delineates in detail the DCE-MRI digital imaging and communications in medicine (DICOM) files with DICOM radiation therapy (RT) structure sets and digital reference objects (DROs), as well as, relevant clinical data that encompass a data set that can be used by all software for comparing quantitative metrics. Variable flip angle (VFA) with six flip angles and DCE-MRI scans with a temporal resolution of 5.5 s were acquired in the axial direction on a 3T MR scanner with a field of view of 25.6 cm, slice thickness of 4 mm, and 256×256 matrix size.

## Background & Summary

Worldwide, head and neck squamous cell carcinoma (HNSCC) is the sixth most common by incidence^1^. Despite advances in treatment techniques, HNSCC 5-year survival has stayed around 60%^2^. Researchers have looked into quantitative metrics that allow individualized risk assessment in order to individualize treatment and improve survival. One quantitative imaging technique, dynamic contrast-enhanced magnetic resonance imaging (DCE-MRI), non-invasively gives measures of the microvasculature within tissue^3^. The metrics from DCE-MRI have been associated with tumor hypoxia which has been linked with poor prognosis for HNSCC^4,5^. Investigations have shown different DCE-MRI metrics to be associated with therapeutic response in HNSCC^6,7^. Additionally, previous studies have found DCE-MRI metrics for HNSCC patients to correlate with salivary gland changes, which can be linked with normal tissue toxicities such as xerostomia^8–11^. Therefore, individualizing treatments for HNSCC may be possible using each patient’s DCE-MRI data.

DCE-MRI has not be used in multi-site clinical trials for HNSCC yet to the best of our knowledge. Before these trials are conducted, DCE-MRI must be thoroughly investigated for reliability across algorithms as differences may impact study results. In theory, the transfer rate constant for contrast from the plasma into the extravascular extracellular space (K^trans^) and the fractional extracellular extravascular space (v_e_) should be independent of scanner, scan protocol, injection dose, and other aspects of the image acquisition. This independence makes DCE-MRI an attractive source for quantitative metrics in prognostic models^12^. Studies have shown that K^trans^ and v_e_ are influenced by the selection of the arterial input function (AIF)^13^, pharmacokinetic model^14^, temporal resolution^15^, and signal-to-noise ratio (SNR)^16^.

Algorithmic implementation of the pharmacokinetic model may vary in aspects such as optimization which can affect the DCE-MRI quantitative metrics. Significant inter-algorithm differences in DCE-MRI metrics have been reported by several studies in the breast, pelvis, and rectum^17–19^. The study by Huang *et al.*^18^ found systematic differences between algorithms in the percentage change in quantitative values through time. The other studies did not find any systematic differences between algorithms^17,19^. Further, Cron *et al.*^20^ demonstrated that larger percentages of unphysical values within regions are computed by algorithms when more noise is present in the DCE-MRI images. The inter-algorithm differences and noise dependence are a hindrance to the clinical implementation of DCE-MRI and prompt thorough investigation towards establishing better repeatability and reproducibility between sites before multi-institutional clinical trials of HNSCC patients with DCE-MRI^21^.

There is a lack of data to use for these investigations into inter-algorithm robustness. Therefore, we have provided 15 HNSCC patients’ DCE-MRI data who were scanned at three time points: before the start of chemoradiotherapy treatment, mid-treatment, and post-treatment. In addition, digital reference objects (DROs) produced by the Quantitative Imaging Biomarkers Alliance are referenced as they have known K^trans^ and v_e_ values, thus can be used as a first check before proceeding to patient data.

Through this data set, we are inviting all researchers interested in quantitative DCE-MRI metrics to investigate the variability in K^trans^ and v_e_ across algorithms. Researchers can do so using digital reference objects (DROs) from the Radiological Society of North America Quantitative Imaging Biomarkers Alliance and the oropharyngeal squamous cell carcinoma patients DCE-MRI scans dataset.

## Methods

### Study population and eligibility criteria

Patients diagnosed with human papillomavirus (HPV)-positive oropharyngeal squamous cell carcinoma were included in this study under a protocol approved by the Institutional Review Board at MD Anderson Cancer Center. All patients gave their study-specific informed consent to participate. The recommendations as described by Freymann *et al.* for de-identification were followed^22^. These recommendations are those from the digital imaging and communications in medicine (DICOM) Working Group 18 Supplement 142 and the Health Insurance Portability and Accountability Act (HIPAA) Privacy Rule^23,24^. Following such standards after patients have signed consent for data for research allows for dissemination of the anonymized patient data.

Patients underwent DCE-MRI scans from December 2013 to October 2014. The criteria for study inclusion were age older than 18 years, histologically documented stage III or IV HPV-positive oropharyngeal squamous cell carcinoma according to the 7th edition of American Joint Committee on Cancer (AJCC) staging criteria, eligibility for definitive chemoradiotherapy, and an Eastern Cooperative Oncology Group (ECOG) performance status of 0 to 2. Of all specified subsites that encompass oropharynx, we opted for the two major subsites of malignant neoplasm of oropharynx (C10) http://www.icd10data.com/ICD10CM/Codes/C00-D49/C00-C14/C10-/C10): base of tongue and tonsils. We adopted the American ICD-10-CM version.

Patients were excluded for any of the following reasons: definitive resection of a primary tumor or administration of induction chemotherapy before radiotherapy, a prior cancer diagnosis except that of appropriately treated localized epithelial skin cancer or cervical cancer, prior radiotherapy to the head and neck, contraindications for gadolinium-based contrast agents, and claustrophobia.

### Patient demographics and clinical end points

Fifteen patients were included in this study. Their median age was 56 years (range, 47–68), with 14 males and 1 female. All patients received radiotherapy at 70 Gy in 33 fractions. The majority of the patients (87%) received cisplatin-based chemotherapy concurrently with radiotherapy. Patient, disease, and treatment characteristics are listed in Table 1. One patient did not have a primary tumor because he or she underwent bilateral tonsillectomy before scanning. Table 2 contains additional information about the clinical information that is provided.

The patients’ demographic data provided include: sex, age at diagnosis and race/ethnicity. Disease characteristics encompassed: oropharyngeal subsite of origin. Furthermore, TNM (tumor, node, and metastases) classification was also provided, where **T category** describes the original (primary) tumor, as regards its size and extent, per the American Joint Committee on Caner (AJCC) and Union for International Cancer Control (UICC) cancer staging system, 7th edition^25^ (https://cancerstaging.org/references-tools/Pages/What-is-Cancer-Staging.aspx). Similarly, the **N category** describes whether or not the cancer has reached nearby lymph nodes, per the AJCC and UICC cancer staging system, 7th edition, along with the corresponding AJCC stage. The addition of **systemic treatment** (whether cytotoxic ‘cisplatin’ or targeted ‘cetuximab’) concurrently with radiotherapy was reported.

### Treatment strategy

Multidisciplinary schematic treatment approach was meticulously detailed by Garden *et al.*^26^ along with MD Anderson Cancer Center protocols of trials studying the implementation of intensity modulated radiation therapy (IMRT) in locally advanced oropharyngeal cancer, e.g. NCT01893307. (https://clinicaltrials.gov/ct2/show/NCT01893307?term=NCT01893307&rank=1). Assessment of an oropharyngeal tumor starts with a global history and physical examination. Typically, this is followed by nasopharyngolaryngoscopy procedure with biopsies of suspicious zones. The vast majority of patients had contrast-enhanced CT scans of the head and neck performed for the purpose of diagnosis and staging of oropharyngeal cancer, whereas some of them underwent other imaging modalities, like positron emission tomography-computed tomography (PET-CT). An institutional transdisciplinary team adopted the comprehensive management approach for all patients at a tumor board, held on a weekly basis. Surgery was mostly implemented for diagnostic purposes, preceding radiotherapy. The selection of the eligible patients for systemic treatment and the prescribed regimens was determined according to the extent of the disease, performance status and associated comorbidities. Consequently, patients with heavy primary tumor disease burden and/or sizable lymph nodes were routinely assigned concurrent chemoradiation.

All patients underwent a non-contrast CT scan for planning purposes a few days before the initiation of radiation treatment while immobilized in the supine position with full-length thermoplastic mask, bite block with or without an oral stent, and a posterior customized head, neck and shoulder mold for radiotherapy. During our Head and Neck Radiation Oncology Planning and Development Clinic, all patients were examined by at least two radiation oncologists and target volumes were peer-reviewed for quality assurance purposes^27^. Gross tumor plus margins were prescribed a dose of 66 Gy for small volume disease and 70 Gy for more advanced disease, and elective regions received 54–63 Gy. Carefully selected patients with well-lateralized tonsil cancers underwent ipsilateral neck irradiation. Appropriate recommendations from the International Commission on Radiation Units and Measurements were followed^28^.

### HPV detection

All tumors were tested for their HPV status via evaluation of the presence of HPV16 DNA by use of the in situ hybridization-catalyzed signal amplification method for biotinylated probes and/or the expression status of p16 via immunohistochemistry (IHC)^29^. In case of any discordance between HPV16 DNA and p16 testing results was encountered, the p16 status was utilized to attribute HPV status due to the fact that p16 positivity can encompass a larger number of HPV strains than in situ hybridization^30^.

### Imaging characteristics and MRI protocol

All patients underwent DCE-MRI scans within 1 week prior to treatment, 3–4 weeks after the start of treatment, and 6–8 weeks after completion of treatment. The DCE-MRI scans were acquired using a 3.0T Discovery 750 MRI scanner (GE Healthcare) with six-element flex coils and a flat insert table (GE Healthcare). The same immobilization devices (individualized head and shoulder mask, customized head support, and intraoral tongue-immobilizing/swallow-suppressing dental stent) were employed in longitudinal scans to improve image co-registration and reduce interval physiologic motion (e.g., swallowing). This setup design has been described by Ding *et al.* where it was shown that this setup with the dental stent is more reproducible than the traditional setup^31^. This setup has not yet been shown to be reproducible across different institutions due to funding limitations, but the improved reproducibility shown at one institution would likely extend to others.

Thirty slices with a field of view of 25.6 cm and thickness of 4 mm were selected to cover the spatial region encompassing the palatine process region cranially to the cricoid cartilage caudally for all scans. Prior to DCE-MRI, T1 mapping was performed using a total of six variable-flip-angle three-dimensional spoiled gradient recalled echo sequences (flip angles: 2°, 5°, 10°, 15°, 20°, and 25°; repetition time/echo time, 5.5/2.1 ms; effective number of excitations, 0.7 (GE terminology, number of averages=percent sampling * number of signal averages/number of excitations); acquisition resolution, 2 mm×2 mm×4 mm; zero filling interpolation, ×2; scan time, 3 min). The DCE-MRI acquisition consisted of a three-dimensional fast spoiled gradient recalled echo sequence to gain sufficient SNR, contrast, and temporal resolution. The following scan parameters were used: flip angle, 15°; repetition time/echo time, 3.6/1.0 ms; number of excitations, 0.7; acquisition resolution, 2 mm×2 mm×4 mm; zero filling interpolation, ×2; temporal resolution, 5.5 s; number of temporal positions, 56; pixel bandwidth, 326 Hz; acceleration factor, 2; and scan time, 6 min. Gadopentetate dimeglumine (Magnevist; Bayer HealthCare Pharmaceuticals) was administered to the patients (dose, 0.1 mmol/kg) followed by a 20-ml saline flush via a power injector (Spectris MR Injector; Medrad) at a rate of 3 ml/second.

A separate AIF was created for 10 patients from an ROI drawn in the following locations: left/right internal carotid artery, left/right vertebral artery. The AIF that fit the most patients was chosen as the population AIF. This was from the first patient’s internal carotid artery. This patient was not included in the database because the field of view was different than those included. Definition of key terms associated with AIF data is tabulated in Table 3.

### Manual segmentation of regions of interest (ROIs) and subsequent analysis

Each patient had 6 ROIs—contralateral and ipsilateral parotid glands, contralateral and ipsilateral submandibular glands, sublingual glands (segmented as one ROI), and primary gross tumor volume—contoured on his or her pre-treatment T1-weighted image by a radiation oncologist with 7 years of experience (A.S.R. Mohamed). Salivary glands were segmented per international consensus guidelines for delineating organs at risk (OARs) for head and neck radiotherapy for research purposes^32^. Gross tumor volumes were defined as per ICRU 62/83, specifically, ‘the gross demonstrable extent and location of the tumor’^28^.

### Data de-identification

We used an in-house code developed in Python (version 2.7) to anonymize the patient DICOM files. This program is designed to replace the patient tags in all the DICOM files in a folder (and sub-folders) with anonymized strings assigned. This was done in accordance with the HIPAA, as designated by the DICOM standards committee Attribute Confidentiality Profile (DICOM PS 3.15: Appendix E), which describes a standard procedure and documentation for removal of protected health information from DICOM images^33^. A final DICOM de-identification quality assurance was applied using a software, ImageJ (https://imagej.nih.gov/ij/), which collects attributes per patient in a report that was scanned to guarantee optimal anonymization accomplishment by the implemented DICOM anonymizer software. To authenticate that our anonymization process hasn’t affected the spatial information included in the DICOM header, we uploaded the anonymized DICOM files into VelocityAI™ 3.0.1 software, where they were correctly viewed.

### Code availability

**ImageJ**, a free software offered by the National Institutes of Health, USA, as a public domain Java processing program. The code for the software is accessible at: https://imagej.nih.gov/ij/.

## Data Records

This head and neck data set (*n*=15) encompasses anonymized clinically curated DCE-MRI scans that show primary tumor as well as major salivary glands segmented by an expert radiation oncologist. These images of 15 patients were obtained at three time points that correspond to pre-, mid- and post-treatment time points.

Relevant clinical meta-data files were also provided as.csv sheets, i.e., comma separated values file, which allows data to be saved in a table structured format. CSVs mimic a spreadsheet but with a.**csv** extension (Traditionally they take the form of a text file containing information separated by commas, hence the name). Table 4 details the various data records, along with brief descriptions. These are cited under (Data Citation 1). While the DCE-MRI images followed the standard DICOM format, images are organized by anonymized patient ID number (Patient_ID) and can be cross-referenced against the data table using this identifier.

The data set that includes all the patient data that we share here is arranged into 15 folders, named as ‘Pat’, i.e., patient number, 1 through 15. Underneath each folder, participants can find 4 folders, one for each time point: pre-, mid-, and post-treatment scans, and one for the structures at pre-treatment. Inside each time point folder, participants can find T1 variable flip angle images and DCE-MRI images. The DICOM tag (0018,1314) identifies the flip angle used for that slice in the variable flip angle images. The Series Description is set to DCE or VFA to identify the DCE-MRI scans and the variable flip angle scans, respectively. Additionally, the Study Date is set to January 1, 1990 for the pre-treatment scans, February 2, 1990 for the mid-treatment scans, and March 3, 1990 for the post-treatment scans. The structures folder contains a DICOM-RT file that includes the 6 ROIs contoured on the pre-treatment image. Along with the patient data is a population AIF.

There are several facets about the data that users should be aware of before proceeding in order to best use the data. The data was acquired at 3T so there is likely B1 variation across the field of view. A B1 map was not acquired under the protocol for these patients. Therefore, to alleviate this issue, users should choose regions in the same spatial locations such that the B1 error is consistent. Additionally, the temporal resolution is low which causes under-sampling of the data and can affect the AIF. If users would like to use a different population AIF to fit parameters due to this, the Parker AIF is suggested^34^.

The DROs can be found at https://www.rsna.org/QIDW/. We recommend DRO version 6 for the noiseless DRO and version 9 for the noisy DROs. The DROs are created using the JSim Tofts-Kermode model. Information about how the DROs for each version were produced are available in a pdf along with the data for that version located at https://sites.duke.edu/dblab/qibacontent/. Additional information on the DROs can be found at http://qidw.rsna.org/.

## Technical Validation

- **Pinnacle treatment planning system** (Philips Radiation Oncology Systems, Fitchburg, WI) engages a collapsed cone convolution (CCC) algorithm, for optimal dose calculation.
- **ClinicStation (Electronic Medical Record System)**, a custom-built electronic medical record system by MDACC, that started in 1999 with subsequent significant improvement in 2007 that allowed further new capabilities, such as integrating research data and accessing data from virtually every electronic source within the institution, thus serving a great role in patient care and research. http://www.clinfowiki.org/wiki/index.php/ClinicStation
- **VelocityAI™ 3.0.1 software** (Varian Medical Systems, Palo Alto, CA), our institutionally-adopted contouring platform, was used for segmenting ROIs.

## Usage Notes

All interested researchers are cordially invited to download the DROs and DICOM files along with clinical meta-data tables for subsequent mechanistic analysis, which includes exploring trends in K^trans^ and v_e_ values over time. Semi-quantitative metrics, such as the area under the curve^12^ can be proposed as more robust surrogate metrics for prognostic studies. One of our aims is to further include assessment of the impact of image noise on quantitative metric error. This step as well as efforts like those by the Quantitative Imaging Biomarkers Alliance to standardize DCE-MRI acquisition parameters represent a natural step forward for quality assurance and serve as the foundation for the current quality assurance work used in the present study. Further study is required before DCE-MRI software-derived parameters can be used clinically in head and neck cancer cases.

## Additional information

**How to cite this article:** Hesham E. *et al.* Dynamic contrast-enhanced magnetic resonance imaging for head and neck cancers. *Sci. Data* 5:180008 doi: 10.1038/sdata.2018.8 (2018).

**Publisher’s note:** Springer Nature remains neutral with regard to jurisdictional claims in published maps and institutional affiliations.

## Supplementary Material



## Figures and Tables

**Table 1:** Study patient demographics.

**Patient Number**	**Sex**	**Age (years)**	**Race/Ethnicity**	**Smoking Status**	**Primary Tumor Site**	**TNM Category**	**AJCC Stage**	**Chemotherapy (weekly)**
1	M	52	White	N	Base of tongue	T3N1M0	III	Cisplatin
2	M	53	White	Y	Base of tongue	T2N2aM0	IV	Cetuximab
3	M	60	White	Y	Tonsil	T4N2bM0	IV	Cisplatin
4	M	55	White	Y	Tonsil	T3N2bM0	IV	Cisplatin
5	M	65	White	N	Base of tongue	T2N1M0	III	Cetuximab
6	M	57	Hispanic	Y	Tonsil	T2N2cM0	IV	Cisplatin
7	M	60	White	Y	Base of tongue	T2N2bM0	IV	Cisplatin
8	M	58	Black	Y	Base of tongue	T2N2cM0	IV	Cisplatin
9	M	62	Asian	Y	Tonsil	T4N2cM0	IV	Cisplatin
10	F	48	White	Y	Tonsil	T4N2bM0	IV	Cisplatin
11	M	56	White	N	Tonsil	T2N2cM0	IV	Cisplatin
12	M	68	White	Y	Tonsil	TxN2cM0	IV	Cisplatin
13	M	47	White	N	Tonsil	T3N2bM0	IV	Cisplatin
14	M	47	White	Y	Tonsil	T3N2bM0	IV	Cisplatin
15	M	55	White	N	Base of tongue	T4N2bM0	IV	Cisplatin

**Table 2:** Supplemental information about the clinical data provided for this data set.

**Data category**	**Description**
Patient ID	Numbers_given_randomly_to_the_patient_after_anonymizing_the_DICOM_PHI_tag_(0010,0020;_Patient_ID)
Sex	Patient's sex
Age at diagnosis	Patient's age in years at the time of diagnosis
Race/Ethnicity	American Indian/Alaska Native, Asian, Black, Hispanic, White or NA (Not applicable)
Oropharynx subsite of origin	The subsite of the tumor within the oropharynx, i.e., base of tongue (BOT) or tonsil
T category	The T category describes the original (primary) tumor, as regard its size and extent, per the 7th edition of American Joint Committee on Caner (AJCC) and Union for International Cancer Control (UICC) cancer staging system. It could be Tx, T1, T2, T3, T4. https://cancerstaging.org/references-tools/Pages/What-is-Cancer-Staging.aspx
N category	The N category describes whether or not the cancer has reached nearby lymph nodes, per the AJCC and UICC cancer staging system. It can be N0, N1, N2a, N2b, N2c or N3. https://cancerstaging.org/references-tools/Pages/What-is-Cancer-Staging.aspx
AJCC Stage	AJCC cancer stage. https://cancerstaging.org/references-tools/Pages/What-is-Cancer-Staging.aspx. It can be 0, I, II, III or IV.

**Table 3:** Definitions of fundamental terms associated with AIF.

**Term**	**Definition**
r1	Contrast relaxivity=3.7 (l/mmol-s) for MAGNEVIST in plasma at 3T at 37 degrees^35^
R1	1/T1 the inverse of the T1 value; relaxivity of blood^36^
R1,0	The inverse of the T1 value of arterial blood without contrast agent=1660 ms^36^
C	Contrast time curve

**Table 4:** Description of data records uploaded to the figshare repository of the HPV and local recurrence prediction challenges.

**Data record**	**Description**
Clinical meta-data for DCE-MRI data set.csv	Clinical meta-data for the 15 oropharyngeal cancer patients, representing our cohort. Patients, disease and treatment identifiers are detailed in this *.csv file.
ReadMe_Clinical meta-data.csv	Supplemental information about the headings of the columns in the clinical data file.
Arterial Input Function.csv	Arterial input function (AIF) extracted from DCE-MRI and required for pharmacokinetic modelling of tumors are provided.
