# Erratum: Dynamic contrast-enhanced magnetic resonance imaging for head and neck cancers

**DOI:** 10.1038/sdata.2018.41

**Published:** 2018-03-20

**Authors:** Joint Head and Neck Radiotherapy-MRI Development Cooperative, Hesham Elhalawani, Rachel B. Ger, Abdallah S.R. Mohamed, Musaddiq J. Awan, Yao Ding, Kimberly Li, Xenia J. Fave, Andrew L. Beers, Brandon Driscoll, David A. Hormuth II, Petra J. van Houdt, Renjie He, Shouhao Zhou, Kelsey B. Mathieu, Heng Li, Catherine Coolens, Caroline Chung, James A. Bankson, Wei Huang, Jihong Wang, Vlad C. Sandulache, Stephen Y. Lai, Rebecca M. Howell, R. Jason Stafford, Thomas E. Yankeelov, Uulke A. van der Heide, Steven J. Frank, Daniel P. Barboriak, John D. Hazle, Laurence E. Court, Jayashree Kalpathy-Cramer, Clifton D. Fuller

*Scientific Data* 5:18008 doi: 10.1038/sdata.2018.8 (2018); Published 13 Feb 2018; Updated 20 March 2018.

An additional word was erroneously inserted into the abstract of this Data Descriptor during typesetting. This has now been corrected in the HTML and PDF versions.

